# Stomach-ache and Consumption

**Published:** 1871-04

**Authors:** T. K. Beecher


### STOMACH-ACHE AND CONSUMPTION.

T. K. BEECHER.

Among the earliest of my recollections, are stomach-ache and peppermint. Occasionally, in aggravated cases or when the peppermint was exhausted, paregoric and elixir pro. came to my relief. But when to me, sleeping in the trundle bed, one-half of it under the bed where my father and mother slept, the colic came in the night time, the remedy was neither peppermint, paregoric nor elixir pro.; but kneadings and pokings by my father's finger. And though I dearly loved peppermint when warmed and sweetened, I am obliged to confess that the parental finger brought relief the sooner.

That which he practiced on his little children he had first verified on his own person. For two or three years of his life a dyspeptic, he experimented largely on drugs and diet. Singularly enough (but not at all surprising to well informed physicians, who are accustomed to all manner of vagaries of personal habit among dyspeptics) he found that he could eat boiled pork and cabbage when all things else distressed him. Let dyspeptics make a note of this; for, except in extreme cases they will find that there is something or other somewhere just queer enough to suit the craziness and absurdity of their disordered stomachs.

As a matter of remedy, however, or permanent relief, my father fell into the use of gastric and abdominal shampooings with his own fingers. None of your little surface-chafing rubs, but judicious pokes and kneadings, by which the sulky stomach and balky bowels were punched up to duty.

I do not know whether the good man ever had access to maps of his inward parts by which to guide his shampooings; or whether he had to feel his way, and learn by experiment the motions that give relief. This only I am sure of;—he used to give relief to his boys; and he worked out a great deliverance for himself from that worst of all diseases that assail professional men, protean dyspepsia; and the treatment was as sound in its theory as it was successful in its application.

Of the medicines that are used for affecting the digestive apparatus, nearly or quite all act upon the mucous surfaces of the organs themselves; and as the hand of a laboring man becomes tough by incessant use of pick and shovel, so any surface becomes tough by incessant meddlings. Just so conscience becomes tough under much preaching. Since it is desirable to preserve the skin or mucous surfaces or conscience in a normal and sensitive condition, we must beware of too much labor, too much medicine, and too much preaching.

The use of physics or tonics for any length of time is a very great unwisdom. But if any person reasonably intelligent will call upon a well equipped physician and ask to see plates or small maps of the digestive organs, and trace them through from the esophagus to the anus, he will be able with very little practice to bring relief to himself or to his friends from almost any distress that arises from indigestion or constipation. What is more to the purpose also, dysenteries and chronic diarrhœas usually give warning days beforehand of trouble within, which an intelligent person will heed. Soreness to the touch before it becomes pain or burning may be allayed by helpful shampooings or by washings with pure soft water.

Every mother of a hearty baby knows that inflammation sometimes comes in the crevices of the little fat boy's arms and round his neck; the heartier the little fellow is, the more certain is he in hot and sweaty weather to suffer by these chafings. The remedy is perfect cleanliness, coolness, and if need be the insertion of an innocent powder or soft linen rag to keep the surfaces from irritating each the other.

That which takes place reddening and distressing a little fat baby's neck, quite often takes place in the bowels of over eating or over working or otherwise imprudent men and women, and instead of medicines a little internal coolness and cleanness is all that is needed—a little soft water.

———The lungs too, often take on disease through want of a little care from their owner. In these days there are few that read but know that the lungs are spongy cellular structures; and that the pure air entering by the nostrils (all should breathe through the nostrils if possible) goes down by the windpipe, and should fill each and everyone of these cells, millions in number, at every inspiration.

While in these cells, whose walls are the thinnest tissues in the body, the blood makes acquaintance with the fresh air and is äerated, that is to say oxygenated.

I suppose there not a hundred persons in the city of Elmira, (if we except our boys who race round and yell and play and *pant* in the open air) who fill their lungs FULL of fresh air as often as once a day. Because all these cells are not needed in the gentle and indolent occupations of our life, we fail to use them. They become like unused rooms in a great house. Unused, they by and by become useless. A careful housewife if she be living alone in a castle, where there is room for a hundred guests and servants proportioned, will at least once a week open every room in the castle and "air it," although she uses but two rooms,—her own and her private kitchen.

Men who have no use for the whole of their lungs should nevertheless use the whole of them at least twenty times a day, drawing a deep breath, and holding it just to the margin of discomfort or penumbra of pain. Hold it so a second or two, and the warmth of the body expands the air thus taken in and distends the lungs. Then breathe it out. Walk a little space in the sunshine and fresh air, and take in another breath a little deeper than before; and so on walking a quarter of a mile and breathing perhaps fifteen times to the full capacity of the lungs, *resting between breaths.*

Persons who have not tried this experiment will be surprised, first at the amount of their expectoration, itself showing how their cells had been clogged up with mucus, and second by the delicious exhilaration when the brain receives such a tide of oxygenated blood as it had not known before for months. I have never myself been able to produce by opium, tobacco, coffee, wine or whiskey so positive and so sensible a stimulation upon my brain as I am able to bring about every time I go out in the open air, by merely breathing my lungs full and rejoicing in the sunshine.

But persons who have been leading sedentary lives for years must be careful in trying these experiments; it would not be difficult as to many of my friends, particularly among the ladies, to produce violent hemorrhage of the lungs, by suddenly expanding them to their utmost capacity. The walls are too weak to stand the strain. Such must proceed very cautiously and be careful not to breathe so as to produce the slightest premonition of pain.

I suppose then that if people will learn how to shampoo their stomach and bowels adroitly and if need be, to wash them with clean water; and if we all learn to breathe the fresh air and give it a chance to work its perfect work, we shall escape half the pains that now afflict us and add one-half to the measurse of our lives. We shall give a permanent good-bye to Stomach-ache and Consumption.

---

AN UNPROFITABLE WIFE.—"Wife," said a broker, a few days since, "do you think I shall ever be worth $50,000." "Ain't I worth that to you?" said the confiding spouse. "Y-e-s," said the other half; "but I can't put you out at interest."—*Exchange.*

Not as profitable as many we wot of. We have known them to increase two, and even twelve fold.

---

BADLY MIXED.—A Lafayette, Indiana, man got mixed up with some car wheels, and the doctors were deliberating, at last account, whether to amputate the man to save his sound leg, or to call the whole thing a total loss.

